# Doctoring John Chinaman

**Published:** 1890-04-26

**Authors:** 


### Doctoring John Chinaman.

In a covering decorated with pictures similar to those which adorned the tea-chests of our youth, but representing the Heathen Chinee having his pulse felt, or swallowing pills and potions, we receive the report of the Hangchow Hospital and Medical Training College. The quaintness of the ornamentation is no mere caprice or convention. Reading the report we meet on every page with evidences that although there be many ills to which all flesh is heir, John Chinaman has ills of his own, and meets them in his own way. He is not by any means ungrateful to the Hospital and the foreign doctors who come to his aid in times of trouble. Some nine or ten mystic hieroglyphics, incomprehensible to the western eye, attached to donations of from one to fifty dollars, prove that the natives of Hangchow are prepared to give something for value received; and the interest they may be expected to show in the strange but beneficent institution that has arisen in their midst is assumed in the printing of a brief appeal in Chinese, which is issued on a sheet of rice-paper, and headed by a diagrammatic representation, with a flavour of the willow-pattern plate about it, of patients ascending and descending the hospital stairs.

Yes, there is a good deal that is unique in this report. The list of things immediately wanted, which includes "an artificial leg for a poor patient, and some thousands of old bottles," as well as the funds for building a new dispensary for out-patients, proves that this hospital does not stand on quite the same footing as those at home; and a closer examination of the notes appended by Dr. D. Duncan Main, the physician and chief benefactor of the institution, show that there is much of a special nature in this medical charity of the far East. The very statistics—though two and two make four in Cathay as in Europe—show something new and strange. The out-patients treated during the year 1888 numbered in all 8,594, in the proportion of 5,838 males, to 2,656 females. The number of in-patients was 481 males, and 70 females. But after this follows an item altogether unique, and in which the proportions of the two sexes are more nearly equal—"Suicides, male 62, female 48, total 110." Can we picture any hospital in Britain where suicides are in the proportion of one to five of the indoor patients? Further statistics inform us that besides these, 31 suicides were treated at their own homes. Of those received at the hospital 18 were dead when brought there, and 26 died after their arrival, but 66 were saved. Suicide by poison seems to be an accepted feature of Chinese life. None of the 110 seems to have thought of knife or pistol, and the vast majority of them—103—essayed to seek death by means of opium.

There is something fascinating in opium. To the Chinese it has the merit of being easily obtainable, nor has he the shrinking from it that would affect an Englishman. So many people smoke opium just for the pleasure of it that it seems natural to take it, and thereby let life, which means debt, poverty, friendlessness, or pain, slip away from their languid hold. The Chinese, as we fancy, do not greatly value life; but they must lay some value on peace and happiness in it, for of the 110 suicides no fewer than 66 had sought death because of quarrels with their friends, while 13 had been tempted to this supreme mode of escape by bad treatment from their parents.

In one way or another, opium runs through the whole report. The treatment of opium smokers forms an important part of the work of the Hangchow hospital. In one year 103 were treated as in-patients and 549 as out-patients, besides others who were treated—naturally under less favourable circumstances—at their own homes. Dr. Main's account of the prevalence of this vice is sufficiently depressing. "There are," he says, "nearly 1,000 registered opium dens in the city and suburbs, and it would be hard to find a village within a radius of many miles that does not contain opium smokers. Opium dens are more numerous than tea shops. In many places opium is more easily obtained than tea or tobacco, and it would seem that opium smoking is steadily increasing." With so many opportunities to tempt them, it is not strange that opium eaters who are cured for a time ultimately relapse into the old vicious habit. Dr. Main speaks of patients returning to the refuge he has established five times in seven years to be cured—if cure it can be called which is so little permanent—yet once more. He speaks earnestly of that decay of moral principle, getting more marked as the craving for the drug increases, which is so marked in opium patients; nor is he to be persuaded, as many English residents in the East finally are, that it is a harmless indulgence.

The opium statistics are interesting. The vice seems rarely to be acquired before maturity; at least only three patients were under twenty years of age. Of the remaining hundred the majority—namely, 35—were over twenty and under thirty, 33 were under forty, and 27 under fifty. Evidently opium smoking is a popular indulgence at every stage of maturity. Nor does it seem that any stand is being made against the vice as such. Of these hospital patients who come seeking to be cured of the habit, 89 began it for the pleasure it afforded, as against 14 who were induced to try it for the relief of pain. And only nine sought to give it up from any moral disapproval, and five because they were exhorted by others to do so. Thirteen repented them when they were ill—we are familiar with such phenomena—but 38 gave up the habit for want of money, 22 because they were out of work, and 17 because they were unable to work, which two latter reasons may be regarded as akin to the first. In short, the Chinaman gives up his opium only when he can no longer afford it. This is not a cheering condition of things. We in England are accustomed to associate the use of narcotics with a sedentary habit of life, and it is therefore surprising to find that the largest proportion of patients from any one class belonged to the farmers, who numbered 19. Next to these come 10 shopkeepers, 10 officials, 9 literati, and 6 fishermen. "Literati" have a bad name for such indulgences, even in this country, especially among those who do not know them; but we expected better things of the farmers.

Dr. Main's notes on the other diseases that have come under his cognizance are very interesting. Many of his patients suffered from ague and other forms of malarial fever, which they believed to be the work of four separate and contradictory demons. One of these fanned the patient till he was cold, another blew him hot, a third, in the guise of a horse's head, made his head ache by piercing it with a sharp trident, while the fourth, with the front of a bull, administered a sound thrashing to make his bones ache. There is a good deal to be said for this myth: it does not outrage probability. Skin diseases also are common, thanks, among other things, to the absence of soap and hot water from the list of a Chinaman's toilet requisites, and to the long nails—so tempting for scratching—which the etiquette of the country makes him wear. To the same lack of cleanliness is due the great prevalence of diseases of the eye. Hernia is frequently met with, and though the patients have not yet been persuaded to submit to a radical cure, they are willing to buy trusses, and probably prefer wearing them to the native mode of treatment, which consists in piercing the swelling to let the air out, under the impression that when the air has escaped the renegade bowel will return to its place. Dr. Main's experience of leprosy is that it does not affect the general health, and that arsenic and quinine, with Easton's syrup, give the most satisfactory results in treatment. Altogether he gives a most interesting account of a unique and valuable little hospital.

